# Rivaroxaban after Thrombolysis in Acute Iliofemoral Venous Thrombosis: A Randomized, Open-labeled, Multicenter Trial

**DOI:** 10.1038/s41598-019-56887-w

**Published:** 2019-12-30

**Authors:** Jin Mo Kang, Ki-Hyuk Park, Sanghyun Ahn, Sungsin Cho, Ahram Han, Taeseung Lee, In Mok Jung, Jang Yong Kim, Seung-Kee Min

**Affiliations:** 1 Departments of Surgery, Gachon University Gil Medical center, Incheon, South Korea; 2 Daegu Catholic University Medical Center, Daegu, South Korea; 3 Seoul National University Hospital, Seoul, South Korea; 4 Bundang Seoul National University Hospital, Seoul, South Korea; 5 Seoul Metropolitan Government Seoul National University Boramae Hospital, Seoul, South Korea; 6 Catholic University of Korea Seoul Saint Mary’s Hospital, Seoul, South Korea; 7 Yeouido Saint Mary’s Hospital, Seoul, South Korea

**Keywords:** Cardiovascular biology, Cardiovascular diseases

## Abstract

Recently non-Vitamin K antagonist oral anticoagulants (NOAC) is replacing warfarin for the treatment of deep vein thrombosis (DVT). However, the role of NOAC after thrombolysis of acute iliofeomral DVT (IFDVT) is not yet defined. This randomized clinical trial aimed to compare the safety and efficacy of rivaroxaban versus warfarin after catheter directed thrombolysis of an IFDVT. Patients with acute DVT of both the iliac and the femoral vein (n = 72) were recruited and randomized to either standard anticoagulation (enoxaparin and warfarin, n = 35) or rivaroxaban (n = 37) after successful thrombolysis or mechanical thrombectomy. Primary efficacy outcome was a recurrence of any venous thromboembolism (VTE) within 6 months. Secondary safety outcomes included major bleeding, clinically relevant non-major bleeding (CRNMB), other adverse event, and all-cause mortality. Rate of recurrent VTE were similar in both groups (11.4% versus 12.5%; *p* = 0.94). Major bleeding or CRNMB was less in rivaroxaban group without significance (2.9% versus 9.4%, HR, 0.31; 95% CI, 0.03–2.96; *p* = 0.31). Recurrence-free survival and major bleeding-free survival at 6 months were not different in both groups. After thrombolysis of acute IFDVT, rivaroxaban was as safe and effective as warfarin in preventing DVT recurrence.

## Introduction

Deep venous thrombosis (DVT) is a serious clinical condition that may cause fatal pulmonary embolism or post-thrombotic syndrome (PTS) encompassing socioeconomic cost and morbidity, especially in extensive acute iliofemoral DVT (IFDVT)^1^. The mainstay of treatment of IFDVT is anticoagulation - either with heparin and classical vitamin K antagonist (VKA) or with novel non-vitamin K antagonist oral anticoagulants (NOAC). In acute IFDVT, the removal of the thrombus through catheter-directed thrombolysis (CDT), is an important adjunctive treatment that promptly recanalizes the obstructed vein and preserve valve function. The CaVenT trial^2^ and recent subgroup analysis of the ATTRACT trial^3^ have shown that additional CDT in the early phase of IFDVT has an advantage over anticoagulation alone in terms of the development of severe PTS afterward.

Recently NOAC has replaced warfarin for the treatment of DVT. However, the safety and efficacy of NOAC after CDT of acute IFDVT remains unknown. Although the results from the large scale prospective randomized studies^4–8^ indicate that NOAC is non-inferior in efficacy and may be safer than VKA as a primary treatment in patients with acute venous thromboembolism, this is not proven in patients after CDT as these patient group has been largely excluded in the majority of the trials^5–8^. Hence, the current guidelines recommend that patients managed with CDT be treated with a standard course of conventional anticoagulation after the procedure without specific recommendations made on the type of oral anticoagulants^9,10^.

Rivaroxaban is an orally active direct factor Xa inhibitor, and is effective for the treatment of acute DVT^7,11^. The authors hypothesized that rivaroxaban was as safe and effective as warfarin for the anticoagulation therapy after thrombolysis of acute IFDVT. Thus, the PRAIS study investigators conducted an open-label, multicenter, randomized study to prove this hypothesis.

## Methods

### Study design and oversight

The PRAIS trial is a pilot randomized, open-label, parallel, multicenter study comparing the efficacy and safety of rivaroxaban with standard therapy (enoxaparin and VKA) after successful thrombolysis in patients with acute symptomatic DVT of iliac and femoral vein. The PRAIS investigators had final responsibility for the study design and oversight, treatment protocols, and collection and analysis of the data. The protocol was approved by the institutional review boards of all participating centers (Seoul national university hospital, Gachon University Gil Medical center, Daegu Catholic University Medical Center, Bundang Seoul National University Hospital, Seoul Metropolitan Government Seoul National University Boramae Hospital, Catholic University of Korea Seoul Saint Mary’s Hospital, and Yeouido Saint Mary’s Hospital), and written informed consents were obtained from all patients. The trial was conducted in accordance with the Good Clinical Practice guidelines and the principles set forth in the Declaration of Helsinki. All suspected outcome events were reviewed and verified by all investigators. The trial was conducted according to the protocol provided as the Supplementary File.

The percutaneous CDT procedure was done by each surgeon’s discretion, either by thrombolysis or by aspiration thrombectomy, and with or without a temporary vena cava filter. Completion venography was used as a marker for treatment success. If the venogram showed complete disappearance of thrombus or residual thrombus less than 50% without flow restriction, it was determined as successful CDT. Residual thrombosis more than 50% or with flow restriction was considered unsuccessful CDT, and the patient was excluded from the study.

**Figure 1:**
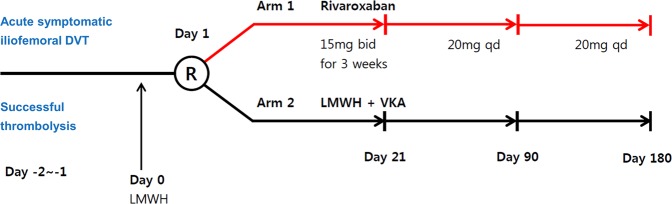
Study design.

After a successful CDT, patients were randomized 1:1 into either the rivaroxaban group or the standard therapy group. The randomization was performed using a web-based randomization program supplied by Medical Research Collaborating Center in Seoul National University Hospital after checking for inclusion and exclusion criteria. Permuted block randomization with blocks of sizes 4 or 6 was used to ensure a balance between the two treatment groups. During the first 24 hours after thrombolysis, all patients received subcutaneous enoxaparin, 1.0 mg per kilogram of body weight twice daily, when randomization was done and any bleeding complication related to the thrombolysis procedure was monitored. (Fig. 1)

The patients randomized to rivaroxaban group received 15 mg twice daily for the initial 3 weeks, followed by 20 mg once daily for 6 months. Patients randomized to standard therapy received enoxaparin and VKA. The target range of international normalized ratio (INR) was 2.0–3.0. Blinding was not possible in the current study because of the different nature of the two medications.

### Sample size

Published recurrence rates of recurrence of DVT in patients with VKA are about 2.0% – 4.0%^4,5,7^. After consultation with the medical research collaboration center in Seoul National University Hospital, the sample size was calculated to perform a non-inferiority trial with a hazard ratio of 0.68, recurrence rate of 3.0%, and a margin of 1.04. With 95% significance and 80% power, the trial needs 4,567 patients, which is almost impossible. Considering the incidence of thrombolytic therapy and the limited number of qualified centers, we conducted this trial as a exploratory study to prove the concept of the idea and decided to enroll 72 patients with 30 patients in each arm and 20% follow-up loss.

### Surveillance and follow-up

Patients were followed at day 21, day 90, and day 180 (Fig. 1). Patient interview and physical examination were performed at each visit, and patients were instructed to immediately report any symptoms of recurrent DVT or bleeding to the study center. CT venography was performed at the last visit. If the patient had a contrast allergy or other contraindication to CT, duplex ultrasonography (DUS) was done.

### Patients

From January 2014 to January 2017, adult patients with acute IFDVT after successful thrombolysis were enrolled in 7 centers in Korea. Patients were included if they were of legal age for consent and had the first episode of objectively verified IFDVT by CT or DUS, and the onset of symptoms within the past 21 days. Patients were excluded if they had remnant thrombus more than 50% or with blood flow restriction on completion venography after the intervention, contraindications to anticoagulation, or VKA treatment within 7 days before enrollment or other indication for vitamin K antagonist. Other exclusion criteria for the current study are listed in Table 1.

**Table 1:** Inclusion and Exclusion criteria.

Inclusion criteria	Exclusion criteria
Patients aged 18–75 years	VKA treatment within 7 days before enrollment or other indication for VKA
IFDVT objectively confirmed by imaging of computed tomography or duplex ultrasound	Creatinine clearance <30 mL/min
The onset of symptoms within the past 21 days	Clinically significant liver disease (e.g., acute hepatitis, chronic active hepatitis, or cirrhosis)
Successful catheter-directed thrombolysis^a^	Liver enzyme level >3X the upper limit of the normal range
Informed consent	Bacterial endocarditis
	Active bleeding or a high risk of bleeding
	Contraindications to anticoagulation
	Systolic blood pressure >180 mm Hg or diastolic blood pressure >110 mm Hg
	Childbearing potential without proper contraceptive measures, pregnancy, or breastfeeding
	Malignant disease needing chemotherapy

Note: ^a^Successful catheter-directed thrombolysis was defined as complete resolution of the thrombus or a residual thrombosis less than 50% without flow disturbance.

### Outcome assessment

The primary efficacy outcome was recurrent venous thromboembolism, defined as the composite of recurrent DVT or pulmonary embolism in 6 months. Recurrent DVT was defined as a new intraluminal filling defect on CT venography or non-compressibility of a previously compressible proximal venous segment on DUS. Acute pulmonary embolism was defined as the presence of a new intraluminal filling defect in pulmonary arteries on a CT scan.

The secondary safety outcome was a composite outcome of major bleeding or clinically relevant non-major bleeding (CRNMB). Major bleeding was defined as clinically overt bleeding or a fall in hemoglobin level of at least 2 g/dL, need for transfusion of two or more units of red blood cells. CRNMB was defined as overt bleeding not meeting the criteria for major bleeding but associated with medical intervention, unscheduled contact with a physician, interruption or discontinuation of protocol treatment, or associated with any other discomfort such as pain or impairment of daily activities.

Other adverse events, including all-cause mortality and vascular events (acute coronary syndrome, ischemic stroke, transient ischemic attack, or systemic embolism), were also analyzed.

### Statistical analyses

All analysis was conducted with the intention-to-treat population. Continuous and interval variables are reported as median values with range. SPSS v17.0 software package (SPSS Inc., Chicago, IL, USA) was used for the statistical analyses. Continuous variables were expressed as mean ± standard deviation, while categorical variables were presented as the percent frequency. Categorical variables were assessed using the chi-square test. A *p*-value of less than 0.05 was considered statistically significant. To compare the outcomes of the two groups, time to event analysis was conducted using Cox proportional hazards regression model to calculate hazard ratios (HRs) and 95% CIs.

### Role of the funding source

The PRAIS study was an investigator-initiated trial and sponsored by Bayer Korea. This trial was registered in ClinicalTrials.gov, number NCT01986192 (date of registration, 18/11/2013). The sponsor of the study had no role in the conduct of the analysis or drafting of the report. All statistical analysis was done by the investigators, and all authors confirmed the accuracy and completeness of the data reported. All authors were involved in the final decision to submit the manuscript.

## Results

### Study enrollment

**Figure 2:**
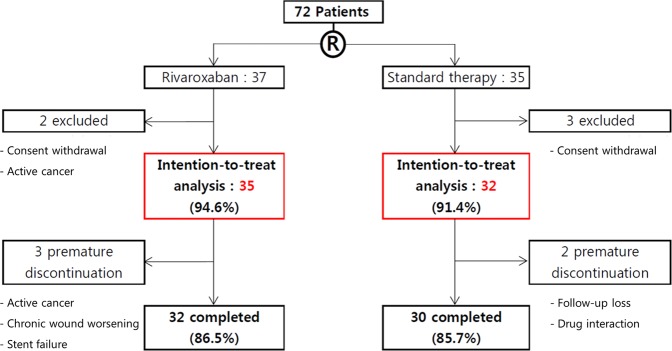
Trial flow.

From January 2014 through January 2017, a total of 72 patients underwent randomization in the PRAIS Study (Fig. 2). A total of 67 patients were included in the intention-to-treat analysis (35 in the rivaroxaban group, and 32 in the standard therapy group). Of these patients, 32 patients (86.5%) in the rivaroxaban group and 30 patients (85.7%) in standard therapy group had completed the trial; the reasons for exclusion and premature discontinuation of both drugs are described in Fig. 2.

### Patient characteristics

Baseline characteristics of patients in both groups are shown in Table 2. There was no statistically significant difference in all variables of baseline characteristics, lesion characteristics, and treatment details. In this study, 85% of patients had left-sided DVT, and 49.3% had May-Thurner syndrome (Table 3). IVC involvement was present in 17.9%, and 37.3% of the patients had concurrent PE. For the treatment, temporary IVC filter was inserted in 50.7% of patients, and the iliac vein was stented in 86.6% (Table 4). IVC filters were usually removed during the index admission or within 2 weeks.

**Table 2:** Baseline characteristics of the patients.

Characteristic	Rivaroxaban (n = 35)	Standard therapy (n = 32)	Total (n = 67)	*p* - value
Age (yr), mean ± SD	57.3 ± 11.1	60.1 ± 10.9	58.7 ± 11.0	0.31
Male, n (%)	15 (42.9)	13 (40.6)	28 (41.8)	0.85
Body mass index (kg/m^2^)	24.5 ± 3.8	24.6 ± 4.2	24.6 ± 7.6	0.98
**Comorbidities, n (%)**				
- Diabetes mellitus	5 (14.3)	0	5 (7.5)	0.05
- Hypertension	9 (25.7)	13 (40.6)	22 (32.8)	0.19
- Ischemic heart disease	1 (2.9)	1 (3.1)	2 (3)	1.00
- Chronic kidney disease	0	1 (3.1)	1 (1.5)	0.49
**Use of antiplatelets, n (%)**				
- Aspirin	3 (8.6)	3 (9.4)	6 (9.0)	1.00
**Risk factors, n (%)**				
- Surgery (3 mo)	6 (17.1)	4 (12.5)	10 (14.9)	0.74
- Trauma (3 mo)	7 (20.0)	2 (6.3)	9 (13.4)	0.15
- Estrogen	2 (5.7)	3 (9.4)	5 (7.5)	0.66
- Infection (6 wk)	2 (5.7)	0	2 (3.0)	0.49
**Drop-out, n (%)**	5 (13.5)	5 (14.3)	10 (14.9)	1.00
- Consent withdrawn	1	3	4 (6)	
- Newly diagnosed cancer	2	0	2 (3.0)	
- Technical failure	1	0	1 (1.5)	
- Drug interaction	0	1	1 (1.5)	
- Abscess aggravation	1	0	1 (1.5)	
- Follow-up loss	0	1	1 (1.5)

**Table 3:** Lesion characteristics.

Characteristic	Rivaroxaban (n = 35)	Standard therapy (n = 32)	Total (n = 67)	*p* - value
Interval between symptom onset to randomization (days), mean ± SD	8.0 ± 9.2	5.3 ± 5.2	6.7 ± 7.6	0.16
Laterality of DVT				0.43
- Both	2 (5.7)	4 (12.5)	6 (9.0)	
- Left	30 (85.7)	27 (84.4)	57 (85.0)	
- Right	3 (8.6)	1 (3.1)	4 (6.0)	
May-Thurner syndrome	16 (45.7)	17 (53.1)	33 (49.3)	0.54
IVC involvement	6 (18.8)	6 (17.1)	12 (17.9)	0.86
Concurrent PE	12 (34.3)	13 (40.6)	25 (37.3)	0.59

Note: DVT, deep vein thrombosis; IVC, inferior vena cava; PE, pulmonary embolism.

**Table 4:** Treatment details.

	Rivaroxaban (n = 35)	Standard therapy (n = 32)	Total (n = 67)	*p* - value
IVC filter insertion, n (%)	18 (51.4)	16 (50.0)	34 (50.7)	0.91
**Adjunctive therapy, n (%)**				
- Stent insertion	28 (80.0)	30 (93.8)	58 (86.6)	0.15
**Thrombus resolution after CDT, n (%)**^**a**^				0.31
- Complete resolution	26 (74.3)	27 (84.4)	53 (79.1)	
- Partial resolution >50%	9 (25.7)	5 (15.6)	14 (20.9)	
**Complication, n (%)**	6 (17.1)	5 (15.6)	11 (16.4)	0.87
- LFT elevation	1 (2.9)	1 (3.1)	2 (3.0)	
- Puncture site pain	0	1 (3.1)	1 (1.5)	
- Dyspnea	1 (2.9)	0	1 (1.5)	
- Ecchymosis	1 (2.9)	1 (3.1)	2 (3.0)	
- Nasal bleeding	0	1 (3.1)	1 (1.5)	
- Technical failure	3 (8.6)	1 (3.1)	4 (6.0)	

Note: ^a^evaluated by completion angiogram. IVC, inferior vena cava; CDT, catheter-directed thrombolysis; LFT, liver function test.

### Clinical outcomes

The clinical outcomes are shown in Table 5. The primary efficacy outcome of recurrent VTE, developed in 4 patients (11.4%) in the rivaroxaban group compared with the 4 patients (12.5%) in the standard therapy group (*p* = 0.94). Only 1 patient in the standard therapy group was symptomatic.

**Table 5:** Clinical outcomes.

	Rivaroxaban (n = 35)	Standard therapy (n = 32)	Hazard Ratio (95% CI)	*p* – value
**Efficacy outcome, n (%)**				
Recurrent DVT	4 (11.4)	4 (12.5)	0.95 (0.24–3.79)	0.94
**Safety outcome, n (%)**				
Major or CRNMB*	1 (2.9)	3 (9.4)	0.31 (0.03–2.96)	0.31
- Major bleeding	0	1 (3.1)		
- CRNMB	1 (2.9)	2 (6.2)		
Any bleeding	3 (8.6)	3 (9.4)	0.94 (0.19–4.68)	0.94
Adverse event	16 (45.7)	14 (43.7)	1.03 (0.50–2.12)	0.93
**Non-compliance, n (%)**	0	2 (6.2)	—	0.22

Note: CRNMB, clinically relevant non-major bleeding.

**Figure 3:**
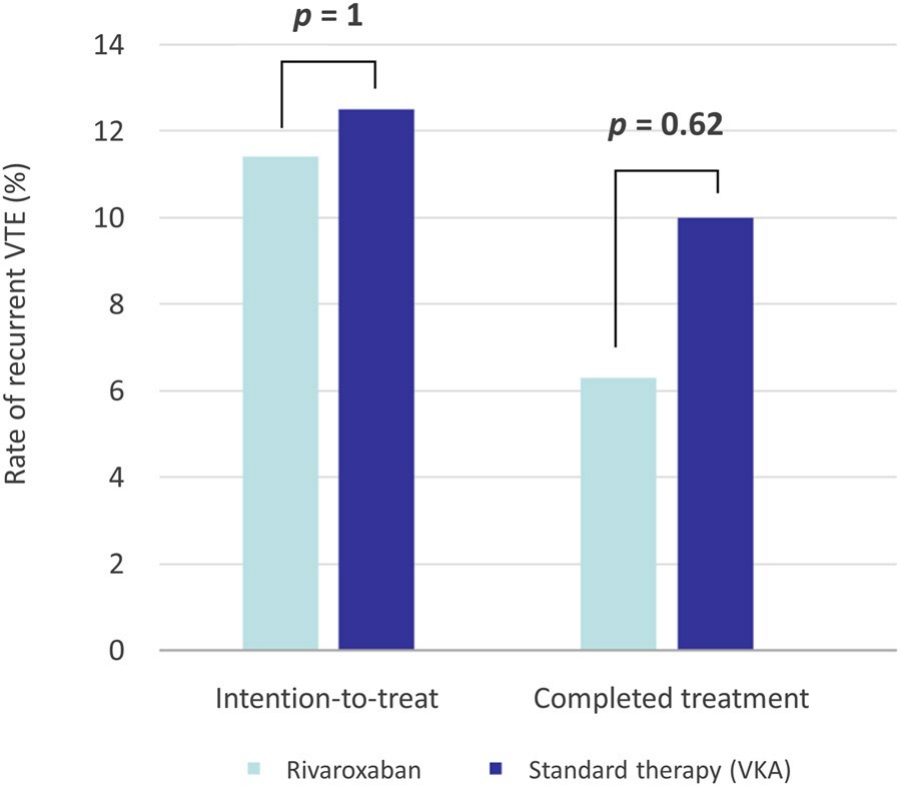
Efficacy outcome of recurrent venous thromboembolic disease at 6 months. Notes: VTE, venous thromboembolism; VKA, vitamin K antagonist.

The efficacy outcome of recurrent venous thromboembolic disease at 6 months is shown in Fig. 3. In the intention-to-treat analysis, recurrent VTE rates were 11.4% in the rivaroxaban group and 12.5% in the standard therapy group. When excluding patients with immediate stent failure during the index admission, the recurrent VTE rates were 6.3% vs. 10%, which was not statistically significant.

There were 4 cases of stent failure defined as early re-thrombosis in the treated segment during the index admission, which was attributed to the technical failure. One patient in the rivaroxaban group was dropped out due to immediate re-thrombosis 1 day after thrombolysis. Actually, the first intervention was a failure but misjudged as a success, and reintervention was also not successful. The other 3 patients underwent re-intervention during the index admission in a single center, and complete resolution of the thrombi was achieved. These patients completed the study and were included in the intention-to-treat analysis.

**Figure 4:**
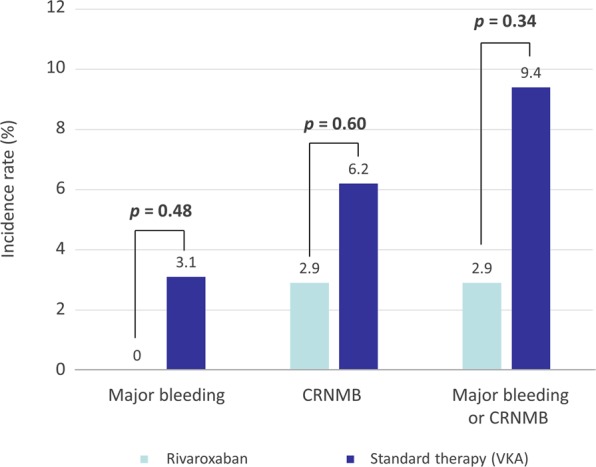
Safety outcome of major bleeding or clinically relevant non-major bleeding.

The safety outcome of major bleeding or CRNMB occurred in 6% of patients (Fig. 4); 1 (2.9%) in the rivaroxaban group and 3 (9.4%) in the standard therapy group (*p* = 0.31). Major bleeding occurred in 1 patient in the standard therapy group (small bowel intraluminal bleeding). Three cases of CRNMB were nasal bleeding (n = 1, rivaroxaban group), hematochezia (n = 1, warfarin group), hematuria (n = 1, warfarin group).

Any bleeding, including clinically non-relevant bleeding developed in 9% (8.6% of the rivaroxaban group and 9.4% of the standard therapy group). Non-compliance was detected only in 2 patients (6.2%) the standard therapy group (*p* = 0.22). Fortunately, all-cause mortality and vascular event did not occur during the study period.

## Discussion

The efficacy and safety of rivaroxaban have been proven by the EINSTEIN DVT trial, which showed that patients with 3–12 months treatment of rivaroxaban had non-inferior efficacy regarding VTE recurrence and bleeding event compared to VKA^7^. However, as in many trials comparing NOAC with VKA, patients treated with fibrinolytic agents for the index episode of DVT were excluded from enrollment in the EINSTEIN trial. Data from the CaVenT and ATTRACT trials comparing anticoagulation alone and CDT with anticoagulation also provided limited information on the role of NOAC after CDT as the study population was treated with VKA predominantly^12^, if not exclusively^2^. However, in real practice, there is a major shift toward the use of NOAC instead of VKA, even after CDT. Milinis *et al*.^13^ reported that in an international Delphi consensus study, one-third of doctors reported the use of NOACs after CDT even though NOACs were not licensed for use in patients with venous stent after thrombolysis. Thus there has been an increasing need for evidence on the role of NOACs in DVT after CDT.

The current study shows that anticoagulation with rivaroxaban is as effective in preventing DVT recurrence as standard anticoagulation with VKA after CDT in IFDVT. In addition, rivaroxaban seems to be as safe as low molecular weight heparin bridged to VKA in terms of bleeding events after CDT. To our knowledge, there has been only one comparative study to date besides the current study. A single-center prospective registry study, recently published by Sebastian *et al*.^14^ enrolled consecutive IFDVT patients who had nitinol stent placed after CDT and compared patients anticoagulated with rivaroxaban (n = 73) to those anticoagulated with VKA afterward (n = 38). There was no difference in the primary and the secondary patency between the rivaroxaban and the VKA group (87% vs. 72% and 85% vs. 94% at 24 months, respectively), and the rate of bleeding complications was also similar (8% vs. 13%). While the results of the study by Sebastian *et al*. supported the effectiveness and safety of rivaroxaban after CDT, the evidence was limited by the unrandomized nature of the study with potential sources of bias (no specific protocol for selecting the type and length of anticoagulation therapy) and baseline imbalance (VKA patients had a longer duration of symptoms before treatment). The current trial, being the first randomized controlled study to compare NOAC and VKA after CDT, complements the previous evidence provided by Sebastian *et al*. and further supports the use of rivaroxaban after CDT.

Our study also demonstrates the safety of CDT. Major bleeding events were rare in both groups (overall 1%). Along with the results of the ATTRACT trial and CaVenT trial (0.8% and 1.6% major bleeding, respectively), this shows that if we carefully perform CDT in selected patients, the risk of bleeding complications can be minimized^15^. Other complications after CDT reported in our study were minor and spontaneously resolved, including 2 cases of liver enzyme elevation and 1 case of puncture site pain, transient dyspnea, and ecchymosis at the puncture site.

CDT followed by adequate anticoagulation in IFDVT was effective in preserving venous flow and preventing thrombus recurrence. The iliofemoral vein patency of the present study was 88.1% at 6 months. The rate is similar to that reported by Sebastian *et al*. (84.7% at 6 months) and superior to those reported in the CaVenT trial (CDT group, 65.9%). Favorable patency outcome observed in our study compared to that in the CaVenT trial may be explained by different rates of iliac venous stenting (current study, 87%; CaVenT trial, 17%). As the usual rates of stent insertion in patients with IFDVT undergoing CDT are 80–95% in European centers, the CaVenT trial has been criticized for the possible insufficient treatment of the causal stenotic lesions^16^. In the current study, stents were liberally inserted if there were collateral flows and residual stenosis associated with extrinsic compression or unlysed thrombus of the iliac vein, to improve the outflow and prevent recurrent VTE. Such active iliac stenting may have improved the patency after thrombolysis.

Some possible limitations of this study need to be mentioned. First, the number of patients is relatively small, and statistical significance was not verified. Sample size calculated to prove the noninferiority of NOAC compared to VKA was over 4,000. Such a large sample size of acute IFDVT patients treated with CDT is challenging to recruit, as there is considerable variability in the current practice among physicians on the treatment of IFDVT (i.e., anticoagulation after CDT vs. anticoagulation alone). This study, therefore, aimed to provide exploratory comparative data on the concept of the idea that NOAC was not harmful compared to VKA after thrombolysis. Second, there were technical differences in the CDT procedure between centers. Although we had only enrolled patients with successful CDT, the specific duration and methods of thrombolysis as well as indications for additional thrombectomy were not standardized and were primarily left to the physician’s discretion. Thus, there is a possibility that flaws in technique may have affected the patency and safety outcomes, however, the overall patency and rates of major bleeding were comparable to those in the previous studies. In addition, the success of CDT was evaluated based on venography, instead of using intravascular ultrasound (IVUS). Recent VIDIO trial has shown that IVUS was more sensitive and accurate than venography in evaluating the degree of iliofemoral venous obstruction^17^. However, the use of IVUS during vascular procedures in Korea is not reimbursed by the National Health Insurance. Lastly, this study enrolled only Korean patients. There are many ethnic differences in VTE incidence and causes, including low incidence of DVT, extremely rare cases of factor V or prothrombin gene mutations in Korean^10^. Thus, these data may not be directly applicable to other ethnicities. We hope new trials with a larger sample size could be done in multi-ethnic groups.

Despite these limitations, this trial has several strengths in methodology. Diagnosis of recurrence was assessed objectively by protocol CTV and not merely dependent on subjective symptoms. Recurrent deep vein thrombosis was defined as a new intraluminal filling defect on CT venography or non-compressibility of a previously compressible proximal venous segment on DUS. The internal validity of both studies was reinforced by the low rate of follow-up loss.

In conclusion, this PRAIS trial shows that rivaroxaban is as effective and safe for patients after CDT due to IFDVT as standard anticoagulation therapy. Rivaroxaban could safely replace VKA after successful thrombolysis for acute IFDVT.
